# Bariatric Surgery and Endoluminal Procedures: IFSO Worldwide Survey 2014

**DOI:** 10.1007/s11695-017-2666-x

**Published:** 2017-04-13

**Authors:** L. Angrisani, A. Santonicola, P. Iovino, A. Vitiello, N. Zundel, H. Buchwald, N. Scopinaro

**Affiliations:** 1 General and Endoscopic Surgery Unit, S. Giovanni Bosco Hospital, Naples, Italy; 2 Gastrointestinal Unit, Department of Medicine and Surgery, University of Salerno, Via S. Allende. Baronissi-, Salerno, Italy; 3 Minimally Invasive and Bariatric Surgery, FSFB, Bogata, Colombia; 4 Departments of Surgery and Biomedical Engineering, University of Minnesota, Minneapolis, MN 55455 USA; 5 Department of Surgery, School of Medicine, University of Genoa, Genoa, Italy

**Keywords:** Bariatric/metabolic surgery, Endoluminal procedures, IFSO survey, Sleeve gastrectomy

## Abstract

**Background and aim:**

Several bariatric surgery worldwide surveys have been previously published to illustrate the evolution of bariatric surgery in the last decades. The aim of this survey is to report an updated overview of all bariatric procedures performed in 2014.For the first time, a special section on endoluminal techniques was added.

**Methods:**

The 2014 International Federation for the Surgery of Obesity and Metabolic Disorders (IFSO) survey form evaluating the number and the type of surgical and endoluminal bariatric procedures was emailed to all IFSO societies. Trend analyses from 2011 to 2014 were also performed.

**Results:**

There were 56/60 (93.3%) responders. The total number of bariatric/metabolic procedures performed in 2014 consisted of 579,517 (97.6%) surgical operations and 14,725 (2.4%) endoluminal procedures. The most commonly performed procedure in the world was sleeve gastrectomy (SG) that reached 45.9%, followed by Roux-en-Y gastric bypass (RYGB) (39.6%), and adjustable gastric banding (AGB) (7.4%). The annual percentage changes from 2013 revealed the increase of SG and decrease of RYGB in all the IFSO regions (USA/Canada, Europe, and Asia/Pacific) with the exception of Latin/South America, where SG decreased and RYGB represented the most frequent procedure.

**Conclusions:**

There was a further increase in the total number of bariatric/metabolic procedures in 2014 and SG is currently the most frequent surgical procedure in the world. This is the first survey that describes the endoluminal procedures, but the accuracy of provided data should be hopefully improved in the next future. We encourage the creation of further national registries and their continuous updates taking into account all new bariatric procedures including the endoscopic procedures that will obtain increasing importance in the near future.

## Introduction

All bariatric procedures currently available are actually considered effective in the treatment of morbid obesity and its related comorbidities compared to non-surgical interventions [1, 2]. The choice of one bariatric procedure over another is generally influenced by a number of factors such as literature results, specific local conditions, and the experience of the surgical staff in each country. Several bariatric surgery worldwide surveys have been previously published [3–6] to illustrate the evolution of bariatric surgery around the world in the last decades. Recently, we have published a global overview describing the number and type of each performed procedure of worldwide bariatric surgery in 2013 [7], together with the trends for the most important procedures during the 2003–2013 decade. Our data showed that sleeve gastrectomy (SG) had a steep increase all around the world, although Roux-en-Y gastric bypass (RYGB) still represented the most performed procedure, while adjustable gastric banding (AGB) declined.

During the last years, different endoluminal procedures (Orbera/BIB, Obalon, Spatz adjustable balloon system, heliosphere bag, primary obesity surgery endolumenal (POSE), stomaphix, Apollo, overstiches, endobarrier) have gained popularity among bariatric surgeons in the attempt to fill the gap between medical and surgical treatment for borderline patients [8].

Our aim in this survey is to report an updated overview of all bariatric procedures performed in the nations of the International Federation for the Surgery of Obesity and Metabolic Disorders (IFSO) in 2014. For the first time, in the 2014 IFSO survey, a special section on endoluminal techniques was added. Moreover, we chose the definition “mini-gastric bypass/one anastomosis gastric bypass (MGB/OAGB)”, as suggested by other authors [9, 10], in an attempt to reduce the heterogeneity in definitions that could generate a loss of accuracy in the reported data.

## Methods

### Survey

The IFSO Secretariat asked the national societies to provide data on the surgical techniques utilized by filling-out the 2014 survey form (Table 1). Each national society returned the data collected from its members, in some cases asking for information from each member of the society, and, in other cases, providing the information directly from a national registry.

**Table 1:**
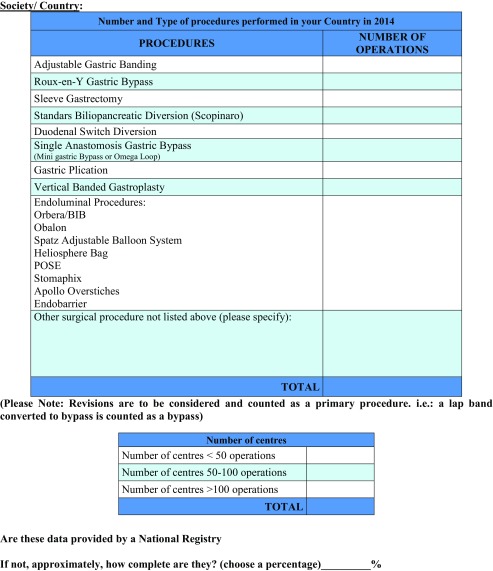
Questionnaire

### Data Analysis

The relative prevalence of specific procedures is provided as weighted averages to account for the wide ranges in the number of procedures performed by the different IFSO member nations or national groupings. These data were used to estimate the annual percentage changes from 2013 [7].

## Results

### Response Rate

**Fig. 1:**
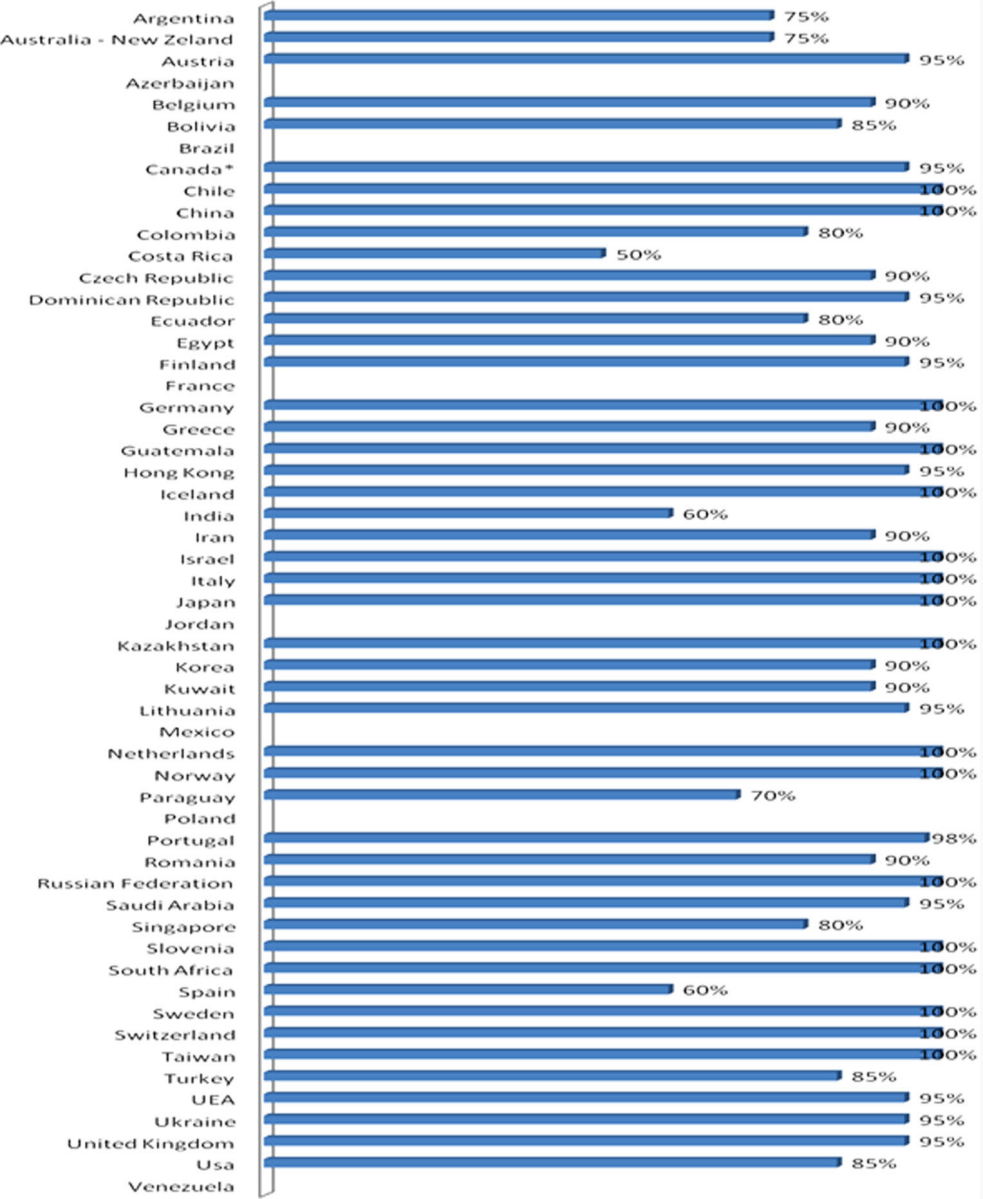
Completeness of data from each responding society

Sixty national bariatric societies or groups were contacted; among them 56 (93.3%) answered and provided a response form. Twenty had a national registry. Figure 1 depicts the completeness of data that each responding society declared, expressed as percentages.

### Number and Type of Procedures

The total number of bariatric/metabolic procedures performed in 2014 consisted of 579,517 (97.6%) surgical operations and 14,725 (2.4%) endoluminal procedures. Tables 2 and 3 show the total number of each bariatric/metabolic surgical procedure together with the percentage of the most commonly performed such as sleeve gastrectomy (SG), Roux-en-Y gastric bypass (RYGB), adjustable gastric banding (AGB), mini-gastric bypass/one anastomosis gastric bypass (MGB/OAGB), biliopancreatic diversion/ duodenal switch (BPD/DS), and the total number of each endoluminal procedure.

**Table 2:** Total number and percentage of bariatric/metabolic surgical procedures performed worldwide in 2014

Procedures	Number	Percentage
Sleeve gastrectomy (SG)	265,898	45.9
Roux-en-Y gastric bypass (RYGB)	229,455	39.6
Adjustable gastric banding (AGB)	42,388	7.4
Mini-gastric bypass/one anastomosis gastric bypass (MGB/OAGB)	10,403	1.8
Biliopancreatic diversion/ duodenal switch (BPD/DS)	6123	1.1
Miscellanea	25,250	4.3
Total	579,517	100

**Table 3:** Total number and percentage of endoluminal procedures performed worldwide in 2014

Procedures	Number	Percentage
Orbera/BIB	1664	11.6
Obalon	741	5.2
Spatz adjustable balloon system	62	0.4
Heliosphere bag	7	0.05
POSE	25	0.2
Apollo	6	0.04
Endobarrier	112	0.8
Not specified	11,658	81.6
Total	14,275	100

Overall total population of the 56 IFSO nations or national groupings in 2014 was estimated as 3,264,082,824 [11], so the 579,517-bariatric/metabolic surgical procedures performed account for 0.02% of the total population.

Table 4 showed the bariatric/metabolic surgical procedures and the endoluminal procedures performed in the four IFSO regions of the world: USA/Canada, Europe, Latin/South America, and Asia/Pacific.

**Table 4:** The bariatric/metabolic surgical procedures and the endoluminal procedures performed in the four IFSO regions of the world: USA/Canada, Europe, Latin/South America, and Asia/Pacific

Country	Total	AGB	RYGB	SG	BPD/DS	GP	MGB/OAGB	Other	Endoluminal procedures
North America									
Canada	6.522	702	3.158	2.362	300	0	0	0	0
U.S.A.	191.920	18.500	59.124	113.381	886	29	0	0	0
Total per area	198.442	19.202	62.282	115.743	1.186	29	0	0	0
Europe									
Austria	2.553	91	1.418	521	4	496	0	23	18
Azerbaijan	16	0	1	14	0	1	0	0	
Belgium	12.000	1.000	5.500	4.000	0	1.500	0	0	
Czech Republic	1.448	280	90	150	50	8	670	200	220
Egypt	10.340	180	1.500	3.100	40	800	200	4.520	1200
Finland	839	0	694	139	4	0	0	2	
France	46.960	4.364	14.015	28.581	0	0	0	0	
Germany	7.298	133	3.332	3.681	9	131	9	3	
Greece	1.327	110	85	756	8	275	65	28	10
Iceland	163	108	52	3	0	0	0	0	
Israel	8881	659	877	7262	65		0	18	
Italy	8867	2182	1628	3799	124	477	268	389	
Lebanon*									
Lithuania	252	63	103	6	0	0	68	12	12
Netherlands	8350	77	6757	1158	10	44	0	304	80
Norway	3002		1653	1316	30	3	0	0	
Poland	2531	318	492	1334	1	207	0	179	179
Portugal	2892	94	1290	986	54	260	9	199	
Romania	1380	27	99	1128	11	22	39	54	17
Russian Federation	1621	419	118	861	71	29	7	116	77
Serbia*									
Slovenia	200	3	31	26	0	115	0	25	
South Africa	566	0	423	63	68	0	0	12	
Spain	4030	126	1562	1839	142	34	27	300	
Sweden	6607	2	5386	1090	47	0	4	78	39
Switzerland	4167	17	3173	646	41	17	0	273	
Turkey	6347	200	1350	3520	125	818	60	274	100
Ukraine	251	2	30	91	47	13	27	41	18
United Kingdom	6391	823	3011	2012	13	0	0	532	198
Total per area	149.279	11.278	54.670	68.082	964	5.250	1.453	7.582	2.168
Latin America									
Argentina	36.668	0	25.520	8.754	2.227	35	0	132	132
Bolivia	314	0	92	174	0	2	36	10	
Brazil	97.480	450	66.000	20.200	1.050	600	30	9.150	8600
Chile	5.311	3	1.133	3.814	0	0	3	358	196
Colombia	12.700	4.800	7.200	0	50	150	0	500	500
Costa Rica	1.448	280	90	150	50	8	670	200	220
Dominican Republic	1.117	0	29	981	7	38	20	42	8
Ecuador	626	0	80	150	3	40	10	343	30
Guatemala	200	2	68	112	0	0	8	10	10
Mexico*									
Panama*									
Paraguay	300	0	300	0	0	0	0	0	
Perù*									
Venezuela	4.472	11	2.880	1.498	3	0	0	80	
Total per area	160.636	5.546	103.392	35.833	3.390	873	777	10.825	9.696
Asia/Pacific									
Australia—New Zeland	15.136	3.604	1.019	10.227	31			255	
China	4.195	50	1.866	2.229	0	0	20	30	
Hong Kong	144	2	4	116	1	1	1	19	17
India	11.336	12	1.833	7.638	28	1.537	12	276	22
Japan	222	4	20	144	54	0	0	0	
Korea	889	439	134	166	0	0	43	107	
Kuwait	5.498	244	61	3.803	4	22	0	1.364	1356
Saudi Arabia	15.571	1.215	3.033	8.649	500	1.580	300	294	294
Singapore	299	2	103	193	0	0	0	1	1
Taiwan	2.421	31	119	1.484	0	194	21	572	34
UEA	4033	170	262	3223	0	230	0	148	87
Total per area	59.744	5.773	8.454	37.872	618	3.564	397	3.066	1.811

*Not received

Nine nations or national grouping reported more than 10,000 bariatric/metabolic surgical procedures: USA (*n* = 191.920), Brazil (*n* = 97.480), France (*n* = 46.960), Argentina (*n* = 36.668), Saudi Arabia (*n* = 15.571), Australia—New Zeeland (*n* = 14.966), Colombia (*n* = 12.700), Belgium (*n* = 12.000) and India (*n* = 11.336). Kuwait’s total population has the world’s highest rate of bariatric/metabolic surgical procedures (0.28%) (Table 5). Kuwait also reported the highest number of endoluminal procedures of the Asia/Pacific Chapter (*n* = 1356). Among the nations of the European Chapter, the highest number of endoluminal interventions was performed in Egypt (*n* = 1200), while in Latin/South America, the largest number was reported by Brazil (*n* = 8600). USA/Canada did not report any endoluminal procedures (Table 4).

### Trends

#### Worldwide

**Fig. 2:**
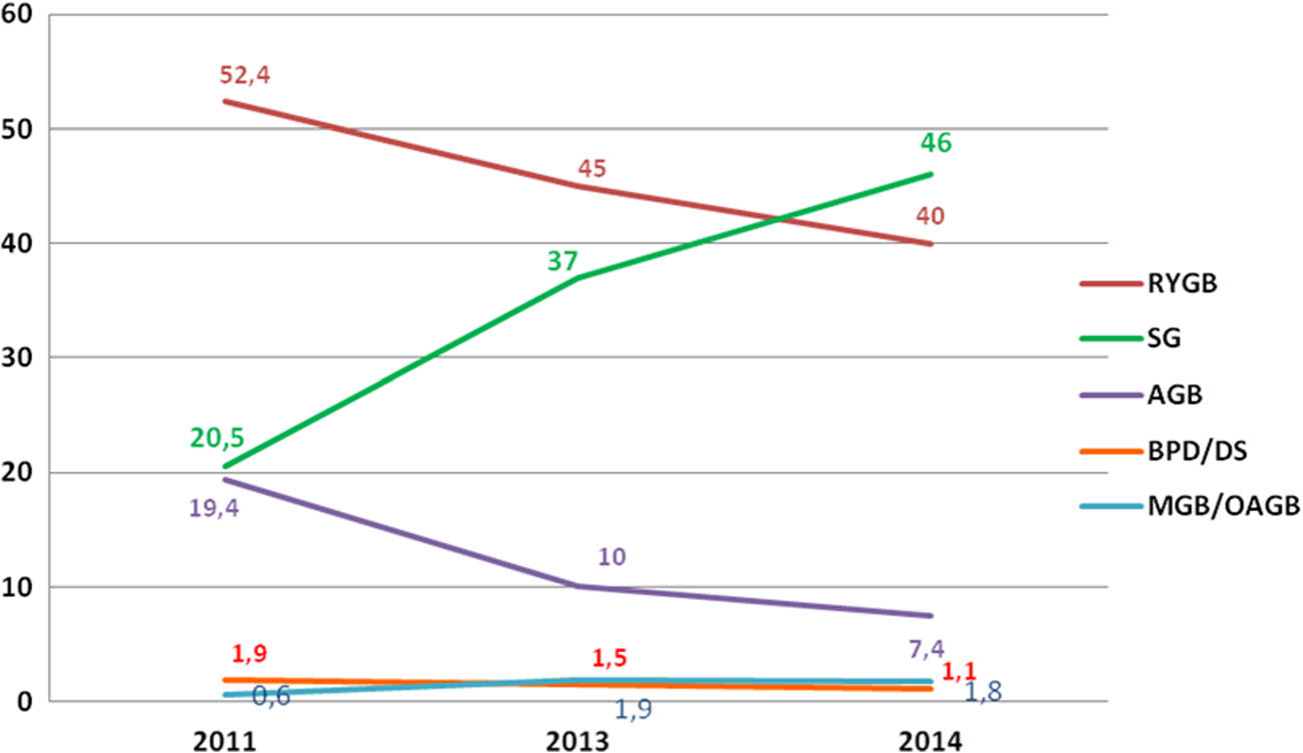
Short-term trend in the world’s main bariatric/metabolic surgical procedures

The annual percentage changes from 2013 of the worldwide bariatric/metabolic surgical procedures revealed that SG had the largest average annual percentage increase of approximately 9%; RYGB and AGB decreased, approximately 5 and 2.6%, respectively. MGB/OAGB and BPD/DS plateaued. Figure 2 presents the short-term trend in the world’s main bariatric/metabolic surgical procedures (SG, RYGB, AGB, MGB/OAGB, and BPD/DS) expressed as the relative proportion at the fixed intervals 2011–2013–2014.

Previous surveys did not report any data on endoluminal procedures, so the annual percentage changes or the time trend for these procedures were not calculated.

#### USA/Canada

**Fig. 3:**
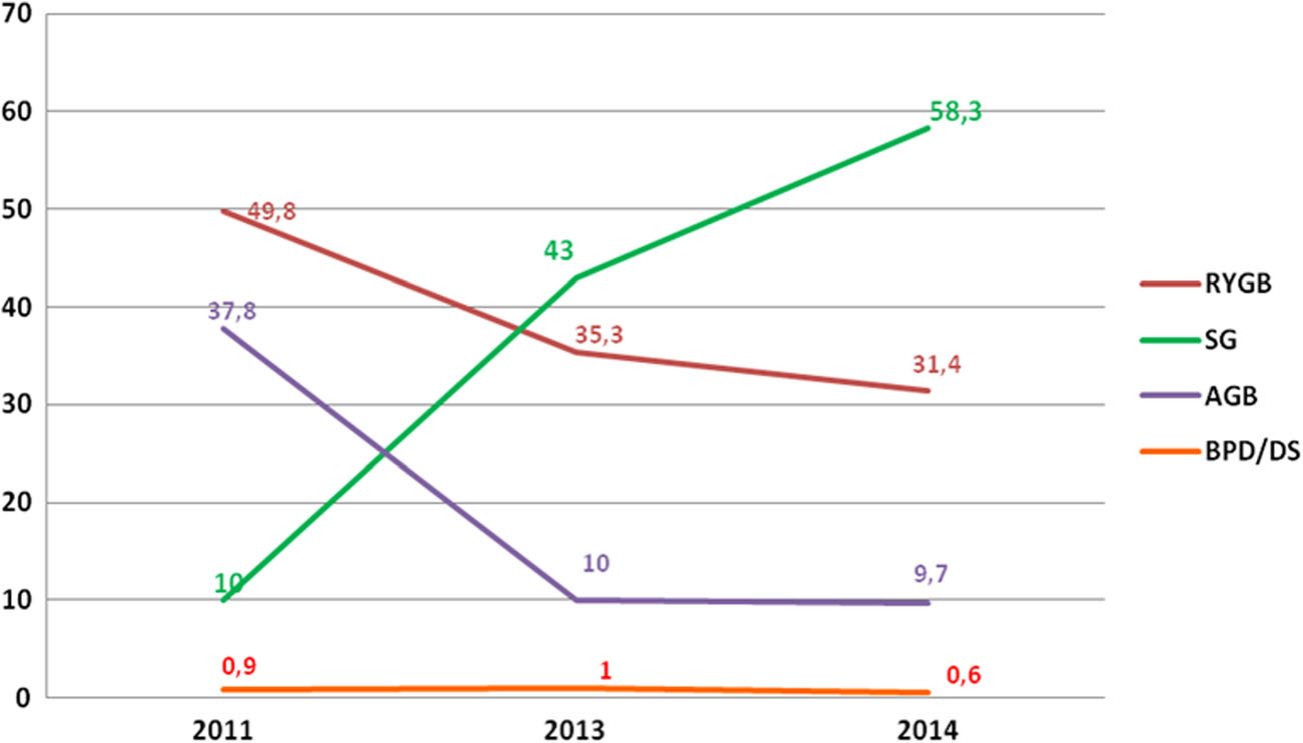
Short-term trend in the USA/Canada of bariatric/metabolic surgical procedures

The annual percentage changes from 2013 in USA/Canada revealed a further increase in the number of SG (+15.3%) that was consistent with the previous survey [7] and the slight decrease of RYGB (−3.9%). AGB and BPD/DS plateaued. Data on MGB/OAGB were not reported. Figure 3 shows the short-term trend of the main bariatric/metabolic surgical procedures (SG, RYGB, AGB, and BPD/DS) in USA/Canada expressed as the relative proportion at the fixed intervals 2011–2013–2014.

#### Europe

**Fig. 4:**
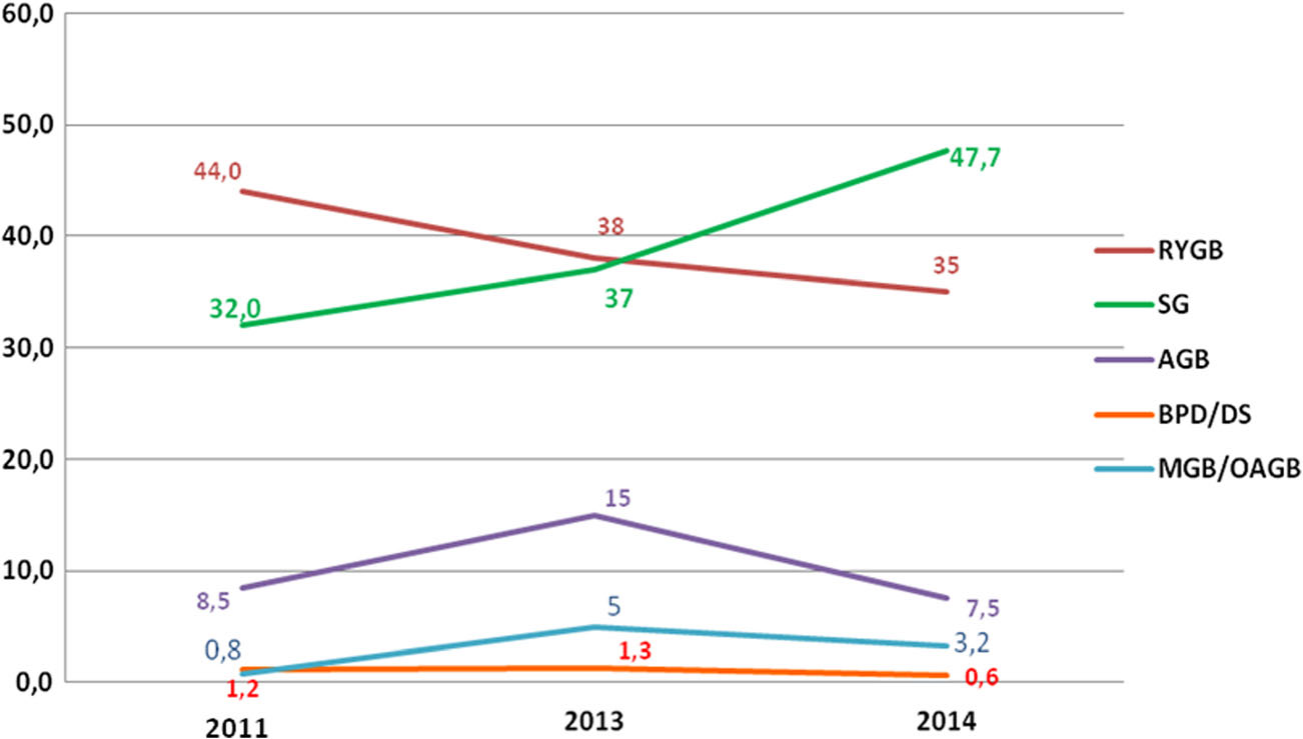
Short-term trend in Europe of bariatric/metabolic surgical procedures

The annual percentage changes from 2013 in Europe revealed a steep increase in SG (+10.7%) and a slight decrease of RYGB (−3%). Also AGB, MGB/OAGB, and BPD/DS decreased (−7.5, −1.8, and −0.7%, respectively). Figure 4 shows the short-term trend of the main bariatric/metabolic surgical procedures (SG, RYGB, AGB, MGB/OAGB, and BPD/DS) in Europe expressed as the relative proportion at the fixed intervals 2011–2013–2014.

#### Latin/South America

**Fig. 5:**
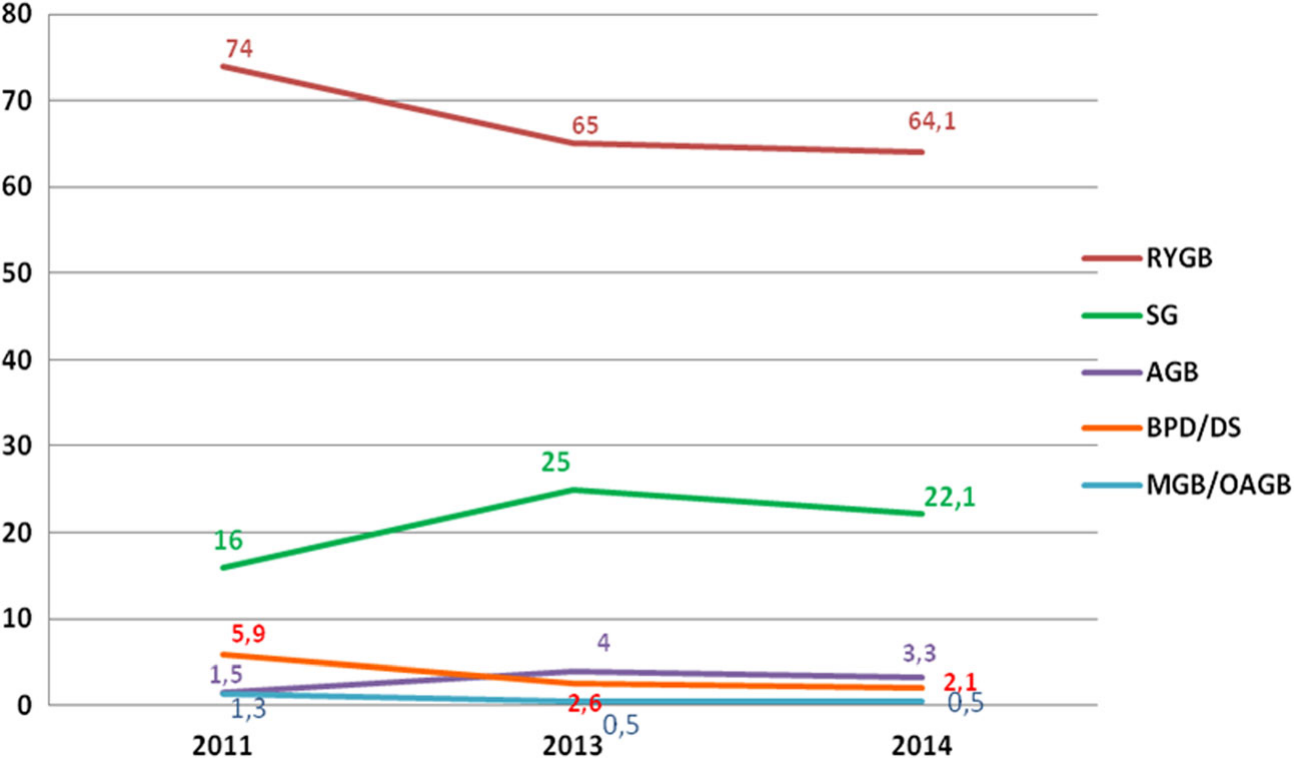
Short-term trend in Latin/South American of bariatric/metabolic surgical procedures

The annual percentage changes from 2013 in Latin/South America revealed that the RYGB plateaued and represented the most frequently performed procedure; SG decreased, approximately 2.9%. AGB, MGB/OAGB and BPD/DS showed a plateauing. Figure 5 shows the short-term trend of the main bariatric/metabolic surgical procedures (SG, RYGB, AGB, MGB/OAGB, and BPD/DS) in Latin/South America expressed as the relative proportion at the fixed intervals 2011–2013–2014.

#### Asia/Pacific

**Fig. 6:**
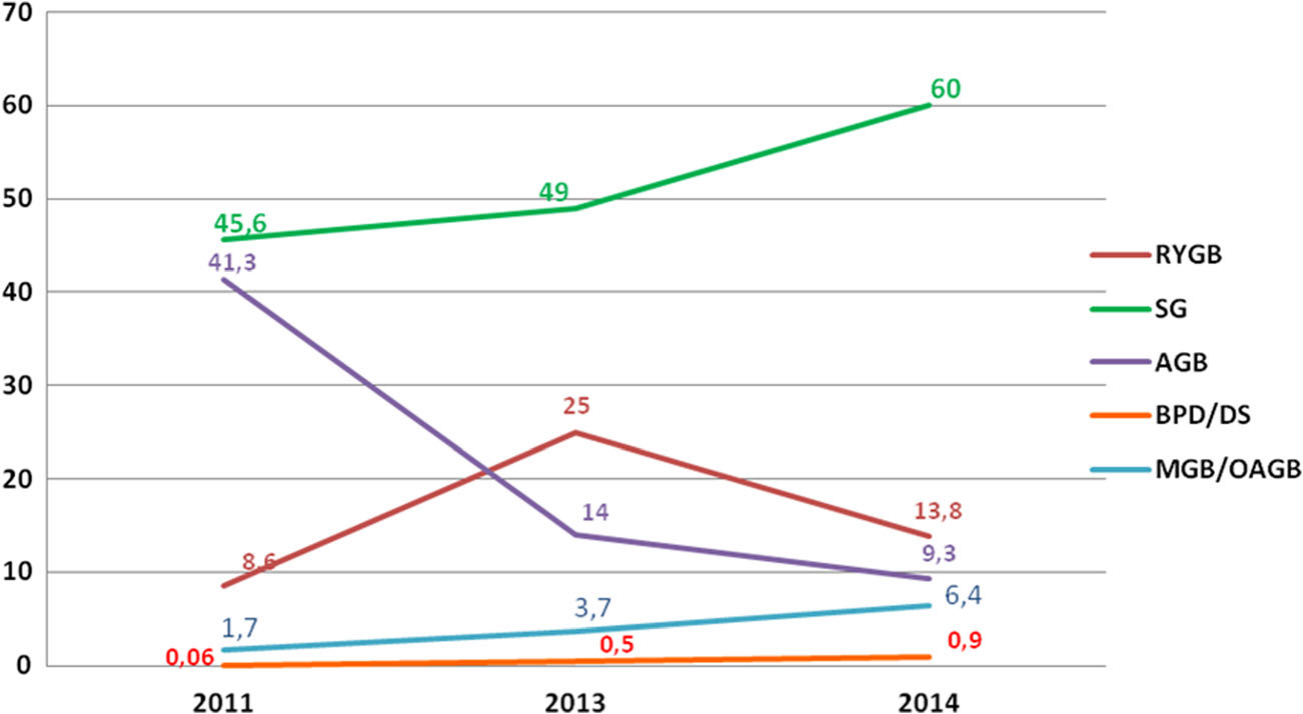
Short-term trend in Asia/Pacific of bariatric/metabolic surgical procedures

The annual percentage changes from 2013 in Asia/Pacific revealed an increase of SG and MGB/OAGB, approximately 11 and 2.7%, respectively; RYGB and AGB decreased (−11.2 and 4.7%, respectively). BPD/DS plateaued. Figure 6 shows the short-term trend of the main bariatric/metabolic surgical procedures (SG, RYGB, AGB, MGB/OAGB, and BPD/DS) in Asia/Pacific expressed as the relative proportion at the fixed intervals 2011–2013–2014.

## Discussion

This survey gives an updated description of bariatric procedures performed worldwide in 2014 and, for the first time, shows the worldwide incidence of endoluminal procedures such as Orbera/BIB, Obalon, Spatz adjustable balloon system, heliosphere bag, POSE, stomaphix, Apollo overstiches, and endobarrier.

This collected data reveal a further worldwide increase in the total number of bariatric/metabolic procedures in 2014 and demonstrate that SG in 2014 became the commonest bariatric procedure performed in the world. The strength of this survey compared to the previous one performed in 2013 was the higher response rate (93.3 vs 90.7%) that demonstrated a further increase of the bariatric/metabolic procedures declared in 2014 (+23%) [7]. Even more interestingly, SG has become the most frequently performed procedure in the world and has overtaken RYGB, which remains the most performed bariatric/metabolic procedure only in Latin/South America. As we have already hypothesized in our previous survey [7] the simpler surgical technique of SG compared to RYGB, together with the promising long-term weight loss outcomes [12, 13], could explain this result.

This survey also shows the short-term trend, from 2011 to 2014, of MGB/OAGB. Rutledge published the first experience on MGB/OAGB in 2001 [14]; it was subsequently performed around the world and several studies supported its efficacy and safety [15]. However, the worldwide MGB/OAGB trend reveals plateauing, with the exception of Asia/Pacific, the only region where MGB/OAGB increased. The current report on the number of MGB/OAGB could be underestimated considering that USA/Canada did not provide any data. Remarkably, the MGB/OAGB prevalence has not been reported not even in the last published estimation of bariatric procedures in the USA carried out by the American Society for Metabolic and Bariatric Surgery (ASMBS) [16].

Another strength of this survey is that we describe for the first time the endoluminal procedures performed in the world. The endoluminal interventions have gained popularity among bariatric surgeons and may be an appealing alternative to a wide group of patients who refuse bariatric surgery because of concerns about potential risks and complications or who were not eligible for bariatric surgery according to the current guidelines. On the other hand, for most of these new technologies, there are currently limited literature data, often based on small series [8] and there are no clinical guidelines. According to our data collection, 14,275 endoluminal procedures have been performed during 2014, but the real number is probably higher. Unfortunately many national databases are still lacking with information on endoluminal procedures. Therefore, we strongly recommend each society to endeavor to report as accurate data as possible.

In an attempt to improve the accuracy of our data, we contacted the manufacturers of the endoluminal devices. They declared a higher number of utilized devices compared to that reported by the IFSO nations. In fact, during 2014, Allergan BioEnterics stated that they have sold 25,043 Orbera/BIB, 953 Apollo endosurgery overstitch, 2935 medical implant helioscopie heliosphere, 5500 POSE, respectively. GI dynamics was not able to provide any data, however, they answered that 2900 endobarriers have been distributed since 2009. Obalon was removed from the market in 2014. Thus, the number of endoluminal procedures performed in 2014 is higher compared to those reported by IFSO nations and we believe that also the number of total bariatric procedures actually performed in the world is greater. Furthermore, the endoscopic and surgical procedures executed in private healthcare were not usually reported.

**Table 5:** Total population and number of procedures per country

Country	Total population	Total procedures per country	% of procedures for total population
North America			
USA—Canada	234,333,465	198.442	*0.08*
Total per area	234,333,465	198.442	*0.08*
EUROPE			
Austria	5,525,965	2.553	0*.*05
Azerbaijan	6,881,963	16	0*.*00
Belgium	6,836,150	12.000	0*.*18
Czech Republic	7,181,452	1.448	0*.*02
Egypt	54,652,669	8.140	0*.*01
Finland	3,393,294	839	0*.*02
France	41,728,824	46.960	0*.*11
Germany	53,375,007	7.296	0*.*01
Greece	7,077,088	1.315	0*.*02
Iceland	212,047	163	0*.*08
Israel	4,886,589	8.869	0*.*18
Italy	40,232,892	8.787	0*.*02
Jordan	4,780,143	7.407	0*.*15
Kazakhstan	12,195,673	114	0*.*00
Lithuania	2,435,496	252	0*.*01
Netherlands	11,059,026	8.350	0*.*08
Norway	3,381,831	3.002	0*.*09
Poland	27,015,538	2.531	0*.*01
Portugal	7,083,260	2.892	0*.*04
Romania	15,224,032	1380	0*.*01
Russian Federation	100,255,437	1.621	0*.*00
Slovenia	1,365,999	200	0*.*01
South Africa	31,673,647	566	0*.*00
Spain	31,954,884	4.030	0*.*01
Sweden	6,148,142	6.607	0*.*11
Switzerland	5,427,608	4.167	0*.*08
Turkey	55,288,903	6.347	0*.*01
Ukraine	30,041,037	251	0*.*00
United Kingdom	34,124,066	6.391	0*.*02
Total per area	611,438,662	154,494,00	0*.*03
Latin America			
Argentina	27,396,887	36.668	0*.*13
Bolivia	13,125,128	314	0*.*00
Brazil	139,204,011	97.480	0*.*07
Chile	12,043,383	5.311	0*.*04
Colombia	31,427,165	12.700	0*.*04
Costa Rica	3,305,826	400	0*.*01
Dominican Republic	6,722,618	1.117	0*.*02
Ecuador	10,117,590	626	0*.*01
Guatemala	8,735,638	200	0*.*00
Mexico	78,705,142	2.500	0*.*00
Paraguay	4,507,926	300	0*.*01
Venezuela	19,024,145	4472	0*.*02
Total per area	354,315,459	162,088,00	0*.*05
Asia/Pacific			
Australia—New Zeland	17,957,192	14.966	0*.*08
China	993,331,831	4.195	0*.*00
Hong Kong	5,205,526	144	0*.*00
India	812,335,761	11.336	0*.*00
Iran	57,467,376	4.919	0*.*01
Japan	77,538,543	222	0*.*00
Korea	35,897,893	889	0*.*00
Kuwait	1,983,536	5498	0*.*28
Saudi Arabia	18,929,961	15.571	0*.*08
Singapore	4,348,692	299	0*.*01
Taiwan	34,593,332	2.421	0*.*01
UAE	4,405,595	4.033	0*.*09
Total per area	2,063,995,238	64,493,00	0*.*00
Total	3,264,082,824	579,517,00	0*.*02

The significance of the value is specified in the first line of the table: it represents the percentage of bariatric procedures/total population (i.e. Among the Austrian population (5,525,965 people) were performed 2553 procedures that represents the 0.05%)

Therefore the accuracy of provided data is the major point of weakness of this survey. Only 35% of national societies had a national registry and most of the data were estimated. This flaw regards the entire database but may be more critical for the endoluminal therapies.

There have been five previous reports of the status of bariatric surgery worldwide [3–7]. All of them, as well as the current survey, were limited by the management and report of the data by the IFSO nations. The analysis of these data can never reach the accuracy and precision of a planned experiment or a clinical trial. However, this is the best achievable analysis of worldwide reported data. Despite these limits, worldwide surveys have always been a scientific landmark. The bariatric surgery community could not progress without a periodic collection and report of worldwide data. Results regarding the techniques of endoluminal bariatric surgery are extremely useful in the current scenario of modern bariatric armamentarium. The reported lack of response aims to be provocative. Data should absolutely be collected at national level by the IFSO societies.

In conclusion, national and international registries should be implemented and regularly updated taking into account all new endoscopic procedures that are continually evolving and will gain increasing importance in the near future. Moreover, further studies on large series of patients are mandatory to increase our knowledge of endoluminal procedures and to encourage the creation of specific international guidelines.
